# p38 inhibition restores chemosensitivity of tumor cells by disrupting oligomerized breast cancer resistance protein membrane trafficking

**DOI:** 10.1016/j.isci.2025.114359

**Published:** 2025-12-10

**Authors:** Yanhong Pan, Ziyan Zhu, Yunxuan Zhu, Tongyao Hu, Zhengyu Zhang, Hui Fan, Suyun Yu, Zhonghong Wei, Aiyun Wang, Yin Lu, Wenxing Chen

**Affiliations:** 1 Jiangsu Key Laboratory for Pharmacology and Safety Research of Chinese Materia Medica, School of Pharmacy, Nanjing University of Chinese Medicine, Nanjing 210023, China; 2 Jiangsu Collaborative Innovation Center of Traditional Chinese Medicine (TCM) Prevention and Treatment of Tumor, Nanjing 210023, China; 3 School of Medicine, Nanjing University of Chinese Medicine, Nanjing 210023, China; 4 Jiangsu Joint International Research Laboratory of Chinese Medicine and Regenerative Medicine, Nanjing 210023, China

**Keywords:** Cancer, Molecular network, Protein

## Abstract

Breast cancer resistance protein (ABCG2) significantly contributes to decreased sensitivity of tumor cells to chemotherapy. While ABCG2 inhibitors exist, multidrug resistance remains unresolved due to limited specificity, toxicity, and heterogeneous expression. To overcome this, we sought to identify key upstream regulators. We assessed drug sensitivity and identified ABCG2 as broadly overexpressed across tumor types and negatively correlated with chemosensitivity. Cell lines with higher ABCG2 expression exhibited lower sensitivity to mitoxantrone, topotecan, and doxorubicin and diminished cytotoxic response. Notably, p38 activation strongly correlated with ABCG2-mediated chemoresistance. Inhibiting p38 phosphorylation effectively downregulated ABCG2 expression and oligomerization. This suppression impaired the drug efflux function of ABCG2, significantly enhancing the cytotoxicity of the chemotherapeutics. Mechanistically, p38 regulated the expression and membrane localization of oligomeric ABCG2, essential for its efflux activity. This study highlights p38 as a promising target to overcome ABCG2-mediated multidrug resistance and improve treatment outcomes for drug-resistant tumors.

## Highlights

• p38 acts as an upstream regulator of ABCG2
• p38 inhibition reverses ABCG2-mediated multidrug resistance
• Inhibiting p38 disrupts oligomeric ABCG2 membrane localization and blocks its drug efflux function

## Graphical abstract

**Figure:**
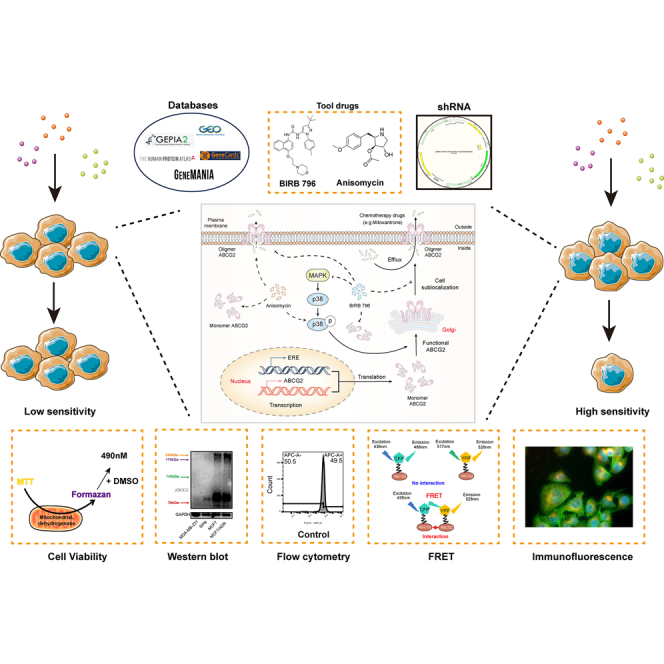

## Introduction

While tumor treatment strategies continue to evolve, chemotherapy remains the mainstay of therapy. However, the emergence of reduced drug sensitivity and multidrug resistance poses a significant challenge to successful cancer treatment.^1^^,^^2^ Many factors lead to the chemotherapeutic sensitivity decrease of tumor cells, including dysregulated apoptosis, autophagy, hypoxia, and DNA damage pathways, with drug efflux pumps representing one of the most prominent contributors. Recently, several studies have discovered the relationship of the ATP-binding cassette (ABC) transporter family with the chemosensitivity of tumor cells, especially P-glycoprotein (P-gp, ABCB1), multidrug-resistance-associated protein 1 (MRP1, ABCC1), and the breast cancer resistance proteins (BCRPs, ABCG2).^3^^,^^4^^,^^5^

ATP hydrolysis induces conformational changes in ABCB1, facilitating efflux of bound hydrophobic drugs into the extracellular space.^6^ While ABCC1 transports similar substrates to ABCB1, it demonstrates limited paclitaxel transport capacity.^7^ Notably, ABCG2 has emerged as a predominant factor in chemoresistance, especially in breast cancer.^8^^,^^9^ Under normal physiological conditions, ABCG2 is primarily located in various tissue barriers, where it plays a crucial role in detoxification processes. However, in the context of tumor cells, ABCG2 often exhibits unfavorable effects.^10^^,^^11^ As a half-transporter, ABCG2 requires dimerization/oligomerization to form functional efflux channels,^12^ a process targeted by cryptotanshinone (CPT), a compound derived from the traditional Chinese medicine *Salvia miltiorrhiza*, which we previously showed could restore mitoxantrone (MIT) sensitivity in MCF7 cells by disrupting ABCG2 oligomerization.^13^^,^^14^ However, current understanding of ABCG2 regulation remains incomplete, with ongoing controversies surrounding its role in chemoresistance and limited data available for solid tumors. Current challenges in ABCG2 inhibition include the need for cell penetration to access ATPase domains, the complexity of its multisubstrate binding sites, and variable expression patterns across malignancies.^15^^,^^16^ They will exhibit inadequate potency when relying solely on substrate competition rather than concurrent efflux blockade. We therefore hypothesized that targeting key upstream signaling pathways, which control ABCG2’s expression, localization, and functional assembly, could represent a more potent and broad-spectrum strategy to reverse chemoresistance. This strategy could achieve broader-spectrum resistance reversal while avoiding the risks associated with excessive inhibition. The mitogen-activated protein kinase (MAPK) pathway, a known regulator of cell proliferation, represents a potential source of upstream ABCG2 regulators. However, existing data reveal a non-uniform picture among different MAPK members. While inhibition of MEK or JNK typically reduces ABCG2 function and enhances chemosensitivity,^17^^,^^18^ the role of p38 remains ambiguous and context-dependent. One research reported that p38 pathway inhibition increased the population of ABCG2-expressing skin epithelial progenitor cells, suggesting a potential negative regulatory relationship in normal cellular contexts.^19^ In contrast, another study demonstrated that pharmacological inhibition of p38 enhanced taxane-induced killing in breast cancer cells by promoting chromosomal instability,^20^ a mechanism that appears independent of ABCG2-mediated efflux. These divergent findings highlight the complexity of the function of p38 and underscore the need to clarify its specific role in regulating ABCG2-mediated drug resistance in tumor cells.

In our study, we discovered that p38 was correlated with reduced sensitivity of tumor cells that overexpress ABCG2 to multiple chemotherapeutic agents. Inhibiting p38 phosphorylation decreased the efflux of chemotherapeutic drugs in tumor cells by downregulating the formation of ABCG2 oligomers on the cell membrane. Our study not only reveals p38 as a potential target that modulates ABCG2-mediated drug efflux but also provides evidence for the application of p38 inhibitors to overcome ABCG2-mediated multidrug resistance in clinical settings, particularly for chemotherapy-refractory tumors exhibiting elevated ABCG2 expression.

## Results

### High ABCG2 expression desensitizes tumor cells to chemotherapy

**Figure 1:**
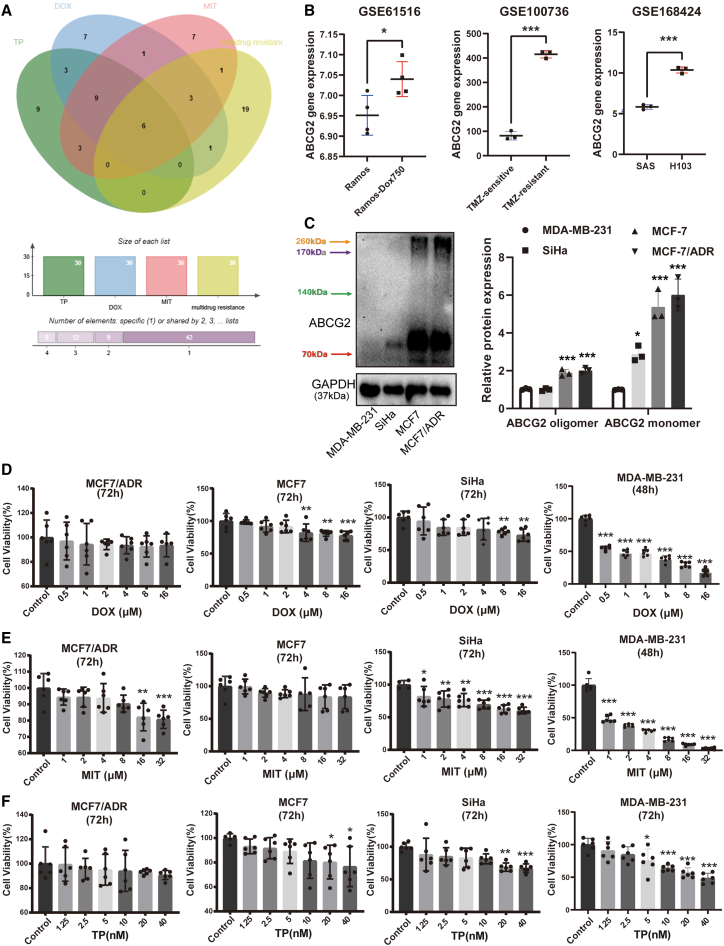
High ABCG2 expression correlates with decreased sensitivity to multiple chemotherapeutic drugs in female reproductive system tumors (A) The Venn map of the top 30 genes related to multidrug resistance, MIT sensitivity, DOX sensitivity, and TP sensitivity. (B) The gene expression of ABCG2 in normal Ramos cells (B lymphocyte) and DOX-resistant Ramos cells (Dox750), TMZ-sensitive cell line and TMZ-resistant cell line (glioma), SAS cell line (oral carcinoma, more responsive to cisplatin treatment), and H103 cell line (oral squamous carcinoma, less responsive to cisplatin treatment). The gene expression values were obtained from NCBI GEO (accession number: GSE61516, GSE100736, GSE1168424). *n* = 4 per group for GSE61516, *n* = 4 per group for GSE100736, GSE1168424. (C) The protein expression and quantitative analysis of ABCG2 in MDA-MB-231 cells, SiHa cells, MCF7 cells, and MCF7/ADR cells. *n* = 3 for each group. (D–F) MCF7/ADR cells, MCF7 cells, and SiHa cells were treated with DOX and MIT at various concentrations for 72h, while MDA-MB-231 cells were treated for 48h. For TP, all cell lines were treated for 72h. The Cell viability was measured using MTT assay. *n* = 6 for each group. Data are mean ± SD, ∗*p* < 0.05, ∗∗*p* < 0.01, ∗∗∗*p* < 0.005.

MIT, doxorubicin (DOX), and topotecan (TP) are widely used chemotherapeutic agents, but their clinical efficacy is frequently compromised by the emergence of drug resistance. To identify shared genetic determinants underlying resistance to these drugs, we performed an integrative bioinformatics analysis. Screening the GeneCards database (www.genecards.org/) for genes related to both multidrug resistance and sensitivity to MIT, DOX, and TP revealed ABCG2 as the most significant candidate among overlapping genes, exhibiting a strong dual association (Figure 1A and Table S1). Gene expression profiling using the Human Protein Atlas database (https://www.proteinatlas.org/) revealed that ABCG2 (vs. ABCB1) is more broadly overexpressed in diverse tumor cell lines, including SiHa, MCF7, A549, and OE19 cells (Figure S1A). Survival from the Kaplan-Meier plotter database (https://www.kmplot.com/) showed significantly worse outcomes in patients with advanced-stage breast cancer (BRCA) and ovarian cancer (OV) exhibiting high ABCG2 expression (Figures S1C and S1D). Moreover, analysis of publicly available data from the GEO database (http://www.ncbi.nlm.nih.gov/geo/) displayed that cell lines resistant to DOX, temozolomide, and cisplatin consistently exhibited elevated ABCG2 expression compared to their sensitive counterparts (Figure 1B). Consistent with these findings, our experimental validation in four tumor cell lines (MDA-MB-231, MCF-7, MCF-7/ADR, and SiHa) demonstrated an inverse correlation between ABCG2 levels and drug sensitivity (Figures 1C–1F and S1D). Specifically, MDA-MB-231 cells, which exhibited the lowest expression of ABCG2, showed the highest sensitivity to all three agents (Figures 1C–1F and S1D). Treatment with 2 μM DOX or MIT for 48 h significantly reduced cell viability in MDA-MB-231 cells, while the same treatments had no significant effect on MCF7/ADR, MCF7, or SiHa cells even after 72-h exposure (Figures 1D and 1E). Similarly, 10 μM TP selectively decreased viability only in MDA-MB-231 cells (Figures 1F and S1D). Compared with the DOX-resistant MCF7/ADR cells, MCF7 cells exhibit greater sensitivity to DOX, MIT, and TP (Figures 1D–1F and S1D). Following a 48-h treatment with 128 μM DOX, 128 μM MIT, and 640 nM TP, the viability of MCF7/ADR cells remained largely unaffected, whereas the viability of MCF7 cells was significantly diminished (Figures 1D–1F and S1D). Among the three cell lines tested (MCF7/ADR, MCF7, and SiHa), SiHa showed higher sensitivity to MIT and TP than both MCF7 and MCF7/ADR cells (Figures 1D–1F). Collectively, these findings indicate ABCG2 as a key mediator of chemoresistance in tumor cells.

### The chemosensitivity of ABCG2-expressing tumor cells is mediated by p38

**Figure 2:**
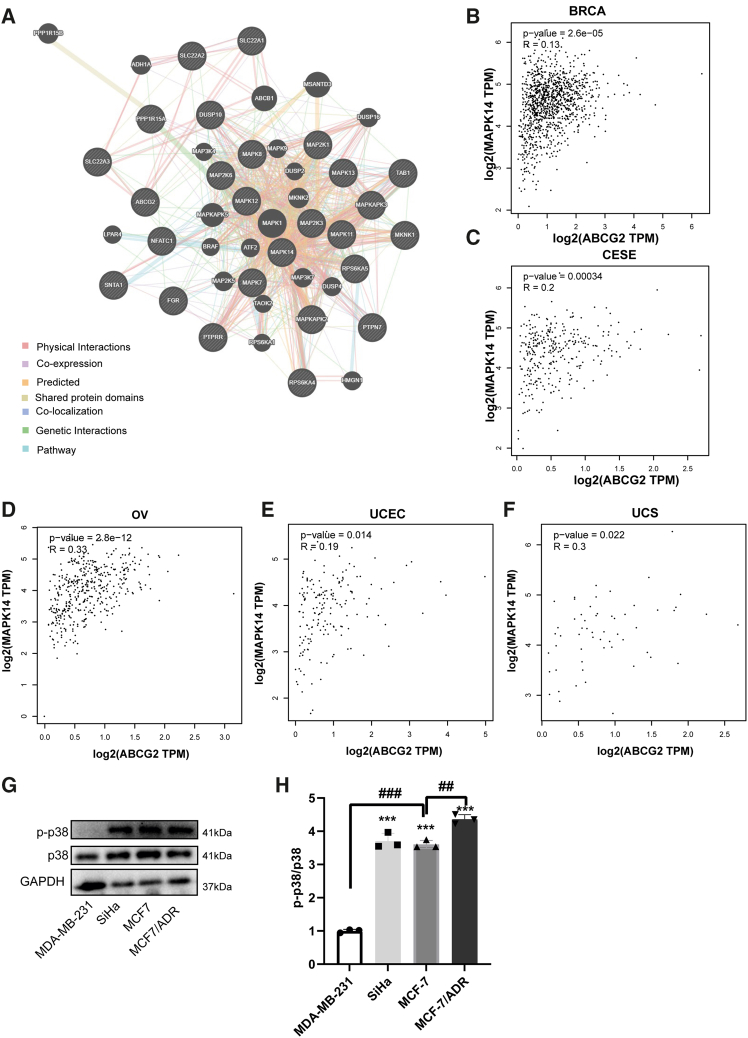
The chemotherapeutic sensitivity of tumor cells expressing ABCG2 is possibly restricted by p38 (A) Protein-protein interaction network of ABCG2 and MAPKs family based on the GeneMANIA database. Positive correlation in the mRNA expression between ABCG2 and MAPK14 gene (encoding p38α) in BRCA (B) and CESE (C), OV(D), UCEC(E), and UCS (F) was obtained from the GEPIA database. (G) The protein expression of p38 and p-p38 in MDA-MB-231 cells, SiHa cells, MCF7 cells, and MCF7/ADR cells. (H)The relative expression level was quantified by ImageJ software. *n* = 3 for each group. Data are mean ± SD. ∗*p* < 0.05, ∗∗*p* < 0.01, ∗∗∗*p* < 0.005, vs. MDA-MB-231 cells. ^##^*p* < 0.01, ^###^*p* < 0.005, vs. MCF7 cells.

Nevertheless, the regulatory network on ABCG2 has not been fully clarified, given the role of the MAPK signaling pathway in tumor growth and drug resistance.^21^^,^^22^ We investigated whether the MAPK/p38 pathway is involved in the regulation of ABCG2 function. Our analysis of the GENEMANIA database (https://genemania.org/) revealed that ABCG2 does not have a direct interaction with p38/MAPK14, but it exhibits genetic interactions with downstream genes of p38/MAPK14, such as MAPKAPK3 and ATF2 (Figure 2A). Using the Gene Expression Profiling Interactive Analysis database (http://gepia2.cancer-pku.cn/), we identified correlations between ABCG2 and genes involved in the MAPK pathway, which may interact with ABCG2 (Table S2). We discovered that p38/MAPK14 was one of the most strongly correlated genes with ABCG2 in patients with BRCA, OV, cervical squamous cell carcinoma, and endocervical adenocarcinoma, uterine corpus endometrial carcinoma, and uterine carcinosarcoma (Table S2). A significantly positive correlation was found between p38/MAPK14 and ABCG2 in these five carcinomas (Figures 2B–2F). In SiHa, MCF7, and MCF7/ADR cells, which are insensitive to MIT, DOX, and TP, high expression of ABCG2 was accompanied by significant activation of p38 (Figures 1C, 2G, and 2H). Especially in breast tumor cell lines, the level of p38 activation was negatively correlated with the sensitivity of tumor cells to chemotherapeutic agents (Figures 1D–1F, 2G, and 2H). Therefore, we speculated that p38 might contribute to the reduced sensitivity of tumor cells to a variety of chemotherapeutic drugs through ABCG2.

### p38 inhibition restores chemosensitivity by blocking ABCG2-mediated drug efflux

**Figure 3:**
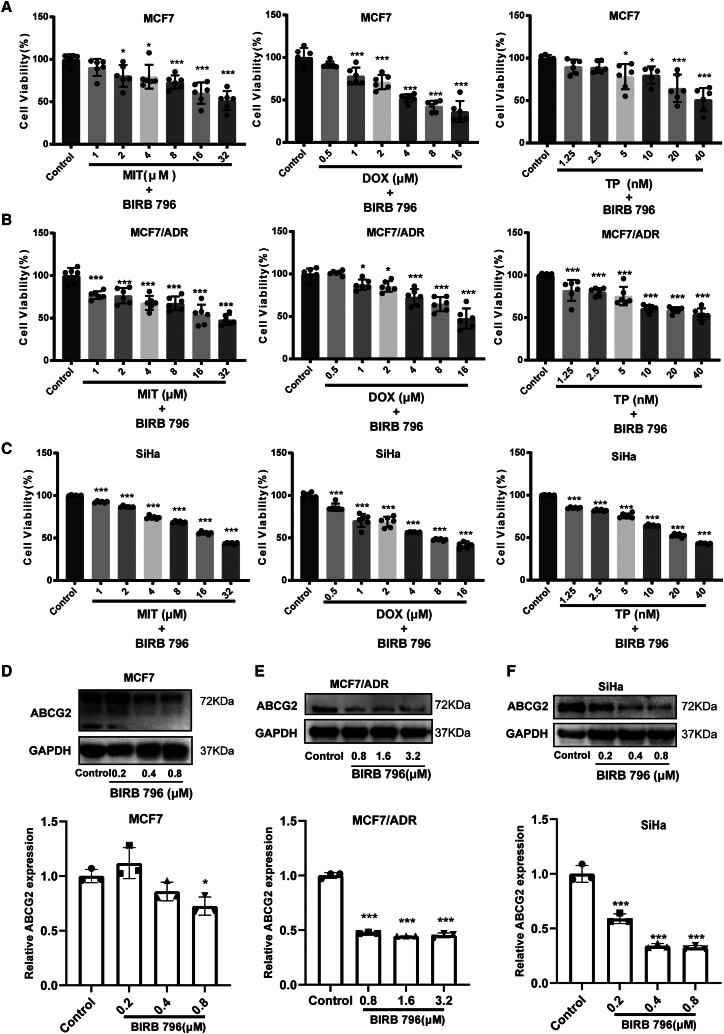
The p38 inhibitor BIRB 796 reverses ABCG2-associated multidrug resistance (A–C) Effect of 1.6 μM BIRB 796 on cell viability of MCF7 cells, MCF7/ADR cells, and SiHa cells treated with MIT or DOX or TP for 72h. *n* = 6 for each group. (D–F) The protein expressions of ABCG2 in MCF7 cells, MCF7/ADR cells, and SiHa cells were determined after BIRB 796 treatment. Relative monomer ABCG2 expression was standardized with GAPDH housekeeping protein expression. *n* = 3 for each group. Data are mean ± SD, ∗*p* < 0.05, ∗∗*p* < 0.01, ∗∗∗*p* < 0.005.

**Figure 4:**
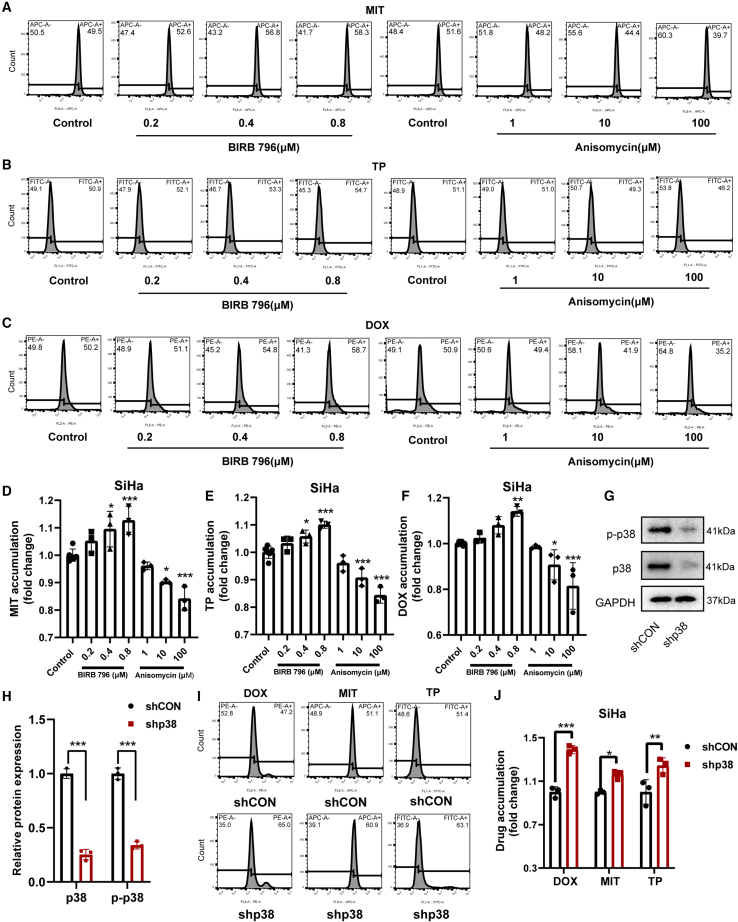
Inhibition of p38 reduces the efflux of tumor cells to multiple chemotherapeutic drugs SiHa cells were treated with MIT (A), TP (B), and DOX (C) after 1h of use of anisomycin or BIRB 796. The drug fluorescence accumulation was detected by flow cytometry. APC-A, PE-A, and FITC-A represented MIT, DOX, and TP respectively. *n* = 3 for each group. The relative accumulation of drugs is shown in Figure (D–F). After shCON or shp38 treatment, (G and H) the expression of p38 and p-p38 proteins and (I and J) the efflux of MIT, TP, and DOX in SiHa cells were measured. *n* = 3 for each group. Data are mean ± SD, ∗*p* < 0.05, ∗∗*p* < 0.01, ∗∗∗*p* < 0.005.

To verify the regulatory role of p38 in ABCG2 function, we employed the p38 inhibitor BIRB 796 to assess its impact on the sensitivity of tumor cells to various chemotherapeutic drugs, including MIT, DOX, and TP, all of which are ABCG2 substrates.^15^^,^^23^^,^^24^ We found that BIRB 796 significantly enhanced the sensitivity of MCF7, MCF7/ADR, and SiHa cells to MIT, DOX, and TP (Figures 1D–1F and 3A–3C). Specifically, 1 μM DOX and 10 μM TP failed to inhibit the viability of MCF-7, MCF-7/ADR, and SiHa cells, but BIRB 796 significantly reduced viability at these doses (Figures 1D, 1F, and 3A–3C). Likewise, 8 μM MIT did not affect MCF-7 and MCF-7/ADR cells, yet BIRB 796 significantly lowered their viability (Figures 1E, 3A, and 3B). In SiHa cells, BIRB 796 raised the inhibitory rate of 32 μM MIT from 70% to 50% (Figures 1E and 3C). The ABCG2 expression in MCF7, MCF7/ADR, and SiHa cells was all obviously decreased by BIRB 796 (Figures 3D–3F). Transport of substrates is an important function of ABCG2, which has been confirmed to be the main reason for its involvement in the reduced chemotherapy sensitivity. We further investigated the effect of p38 on the efflux of ABCG2 substrates utilizing the p38 inhibitor BIRB 796 and the p38 agonist anisomycin. Our findings indicated that BIRB 796 increased the intracellular content of MIT and TP, while anisomycin decreased the intracellular levels of these drugs in SiHa, MCF7, and MCF7/ADR cells (Figures 4A, 4B, 4D, 4E, S2A, S2C, and S2D). Additionally, the activation of p38 decreased the intracellular DOX levels in SiHa and MCF7 cells, whereas inhibition increased DOX accumulation in these cells, as well as MCF7/ADR cells (Figures 4C, S2B, and S2D). To further corroborate the role of p38 in the regulation of ABCG2 substrate efflux, we used shRNA to silence p38 expression, revealing a significant reduction in the efflux of DOX, MIT, and TP in SiHa cells, with increased intracellular drug accumulation (Figures 4G–4J). Taken together, our results suggested that p38 modulated the tumor cell sensitivity to chemotherapeutic agents by regulating the efflux function of ABCG2.

### p38 governs ABCG2 efflux function by modulating its oligomerization

**Figure 5:**
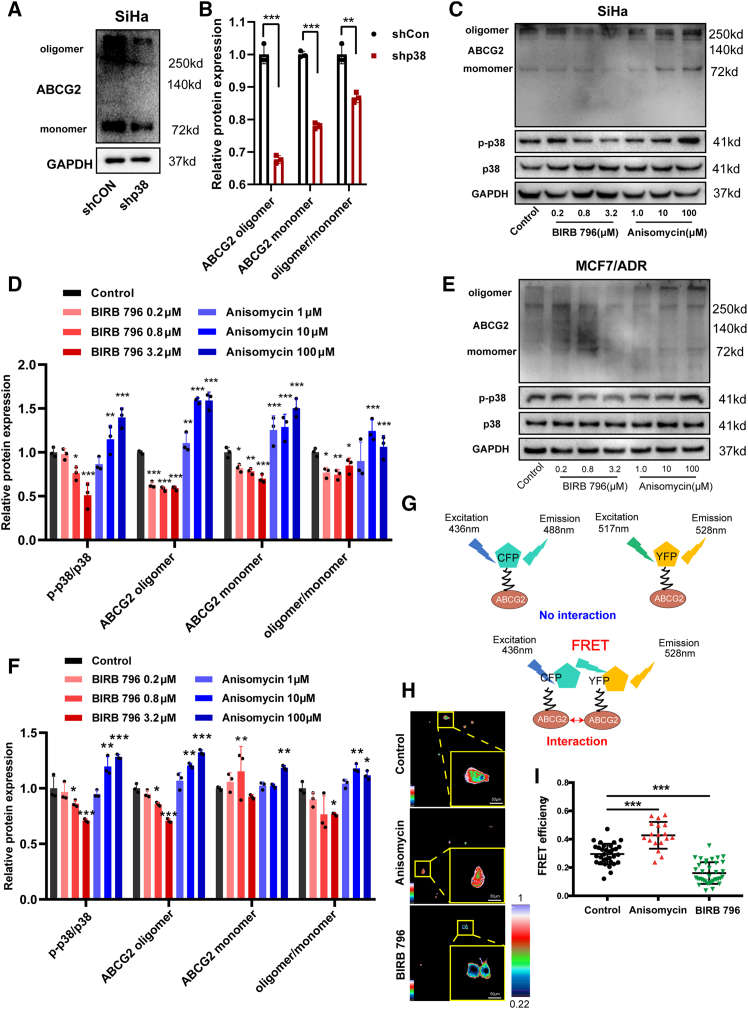
p38 regulates the oligomeric formation of ABCG2 (A and B) The protein expression of ABCG2 in SiHa cells treated with shCON or shp38. *n* = 3 for each group. (C–F) The protein levels of p-p38, p-38, ABCG2 monomer, and ABCG2 oligomer in SiHa cells and MCF7/ADR cells treated with different concentrations of BIRB 796 or anisomycin. *n* = 3 for each group. (G) A schematic diagram illustrating an FRET experiment involving pCFP and pYFP as the fluorescence energy donor and acceptor, respectively, is utilized to measure the oligomer formation of ABCG2 in MCF7/ADR cells treated with 3.2 μM BIRB 796 or 100 μM anisomycin. (H and I) The FRET phenomenon occurs when the distance between pCFP and pYFP is within 10 nm. The color represents the degree of FRET efficiency. Scale bar: 50 μm. *n* = 33 cells for control group, *n* = 18 cells for anisomycin group, and *n* = 36 cells for BIRB 796 group. Data are mean ± SD, ∗∗*p* < 0.01, ∗∗∗*p* < 0.005.

ABCG2 is a semi-transporter, and the formation of its oligomeric structure increases the formation of the outer channel cavity, significantly enhancing the efficiency of efflux substrate drugs.^25^^,^^26^ We investigated the effect of p38 on ABCG2 oligomerization. Compared with shCON SiHa cells, p38-silenced SiHa cells exhibited reduced expression levels of ABCG2 monomer and oligomer (Figures 5A and 5B). Moreover, the ratio of oligomers to monomers decreased significantly after p38 silencing (Figure 5B). Consistent with these findings, treatment with the p38 inhibitor BIRB 796 significantly inhibited the oligomerization of ABCG2 in SiHa cells (Figures 5C and 5D). Conversely, activation of p38 by anisomycin increased the level of oligomerized ABCG2 in SiHa cells, particularly the ratio of oligomeric to monomer (Figures 5C and 5D). Notably, this regulatory role of p38 was not cell-type-specific. Similar effects were observed in MCF7/ADR and MDA-MB-231 cells, where p38 activation enhanced ABCG2 oligomerization and p38 inhibition reduced it (Figures 5E, 5F, S3A, and S3B). In MCF7 cells, while the overall degree of oligomerization remained relatively stable, the expression levels of both monomeric and oligomeric ABCG2 were modulated by p38 pathway activation (Figures S3C and S3D). Specifically, anisomycin treatment increased the expression of both forms, while BIRB 796 treatment decreased it (Figures S3C and S3D). To further prove that p38 regulated ABCG2 oligomer formation, we used the fluorescence resonance energy transfer (FRET) experiment and found that 3.2 μM BIRB 796 significantly reduced FRET efficiency and inhibited ABCG2 dimerization, while 100 μM anisomycin increased FRET efficiency and promoted ABCG2 dimer formation (Figures 5G–5I and S3E). These data indicated that p38 impeded the efflux function of ABCG2 by regulating its oligomeric protein formation.

### p38 disrupts membrane localization to inhibit the efflux function of oligomerized ABCG2

**Figure 6:**
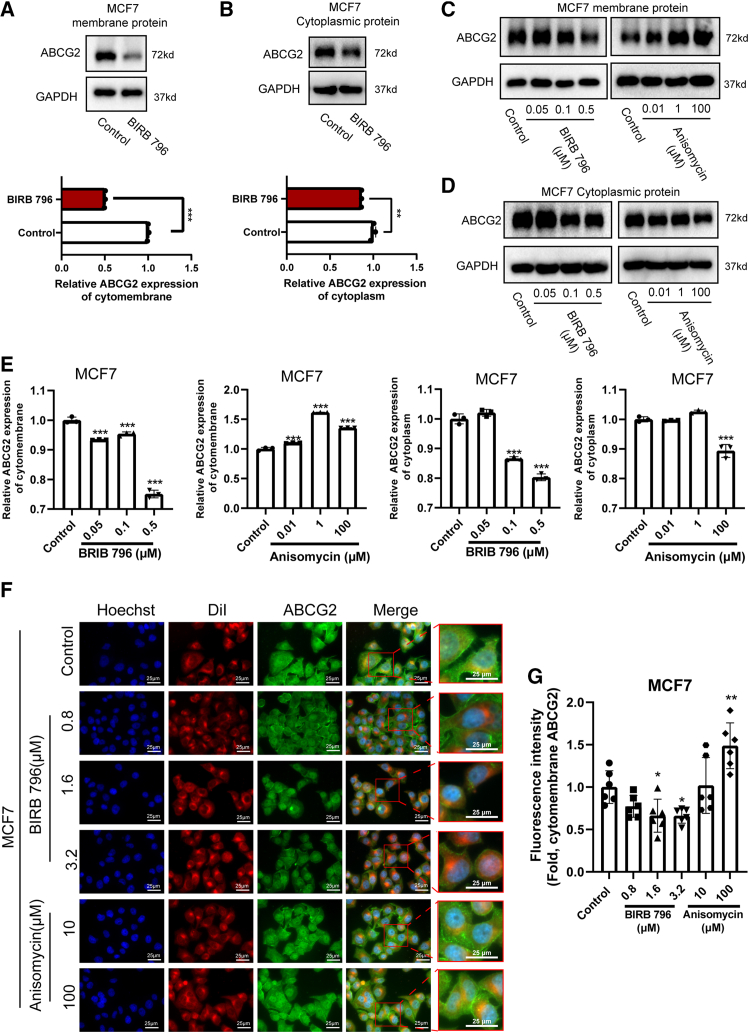
The membrane localization of ABCG2 is regulated by p38 (A–E) Expression of membrane protein and cytoplasmic protein of ABCG2 in MCF7 cells treated with BIRB 796 or anisomycin for 1h. *n* = 3 for each group. (E) The quantitative analysis of ABCG2 protein expression in cell membranes and cytoplasm. (F and G) Immunofluorescence staining for ABCG2 in MCF7 cells treated with BIRB 796 or anisomycin for 1h. Dil was used to indicate cell membrane. Scale bar: 50 μm. *n* = 6 for each group. Data are mean ± SD, ∗∗*p* < 0.01, ∗∗∗*p* < 0.005.

**Figure 7:**
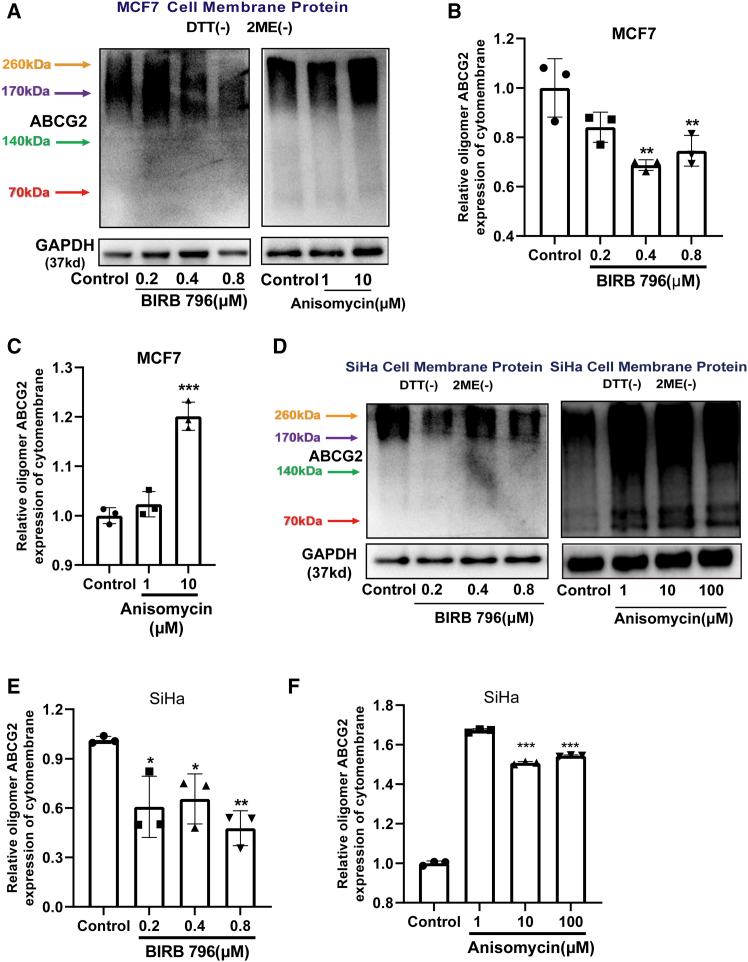
p38 regulates the expression of ABCG2 oligomers on the cell membrane (A) The membrane protein of MCF7 cells was extracted to determine the expression of oligomerized ABCG2 affected by BIRB 796 or anisomycin. The quantitative results are shown in panel (B and C). *n* = 3 for each group. (D) Relative expression of membrane oligomerized ABCG2 in SiHa cells treated with BIRB 796 or anisomycin. *n* = 3 for each group. (E and F) The membrane protein was extracted to determine the expression of oligomerized ABCG2 affected by BIRB 796 or anisomycin. *n* = 3 for each group. Data are mean ± SD, ∗*p* < 0.05, ∗∗*p* < 0.01, ∗∗∗*p* < 0.005.

Oligomer ABCG2 functions as an efflux on the cell membrane, pumping the chemotherapeutic drugs that enter the cell out of the cell. Initially, we examined the expression levels of ABCG2 on the cell membrane and within the cytoplasm of MCF7 cells. Our results showed that inhibition of p38 reduced ABCG2 expression on the cell membrane and in the cytoplasm of MCF7 cells (Figures 6A–6E). Conversely, anisomycin increased ABCG2 expression on the cell membrane while decreased it in the cytoplasm (Figures 6C–6E). When MCF7 and MCF7/ADR cells were treated with BIRB 796, ABCG2 enrichment on their cell membranes was reduced (Figures 6F, 6G, S4A, and S4B). In contrast, anisomycin promoted the enrichment of ABCG2 on the cell membrane (Figures 6F, 6G, S4A, and S4B). A similar effect was observed in SiHa cells (Figures S4C and S4D). Moreover, BIRB 796 decreased the expression of ABCG2 oligomer on the cell membrane, whereas anisomycin increased it (Figures 7A–7F). These results further confirmed that p38 regulated drug efflux through modulating the membrane localization of oligomeric ABCG2, thereby affecting tumor cell chemosensitivity.

## Discussion

The excessive activation of efflux proteins, which significantly diminishes chemotherapy sensitivity, is a major reason for the poor therapeutic outcomes in tumor treatment.^27^ ABCG2, which has garnered attention due to its role in transporting various chemotherapeutic drugs, including MIT, TP, and DOX,^23^^,^^24^ is one such protein. Although research on ABCG2 is not as extensive as that on ABCB1 and ABCC1, it has been shown that ABCG2 has a broad spectrum of substrates, encompassing anthracyclines, camptothecin analogs, methotrexate, and tyrosine kinase inhibitors.^15^ Endogenous ABCG2 expression in tumor cells may contribute to intrinsic drug resistance.^12^ Moreover, its expression levels fluctuate under different pathophysiological conditions and in response to various substances, potentially influencing the bioavailability of therapeutic agents and sometimes exacerbating clinical outcomes.^28^ In our study, drug-insensitive tumor cell lines exhibited high ABCG2 expression. Our previous research revealed that CPT could inhibit the expression and function of ABCG2, with the underlying mechanisms varying depending on differences in ERα expression.^14^ Both our previous and current studies have proved that inhibiting the efflux function of ABCG2 improved the cytotoxic effects of chemotherapy drugs on tumor cells. However, substrate polarity affects the rate of ABCG2-mediated efflux.^29^ Moreover, multiple substrate binding sites of ABCG2 pose a challenge, as reversing resistance to different chemotherapy drugs simultaneously is unattainable with a single ABCG2 inhibitor. Directly inhibiting ABCG2 could lead to severe side effects due to its physiological functions. Instead, targeting key regulators of ABCG2 function might be a more viable solution.

**Figure 8:**
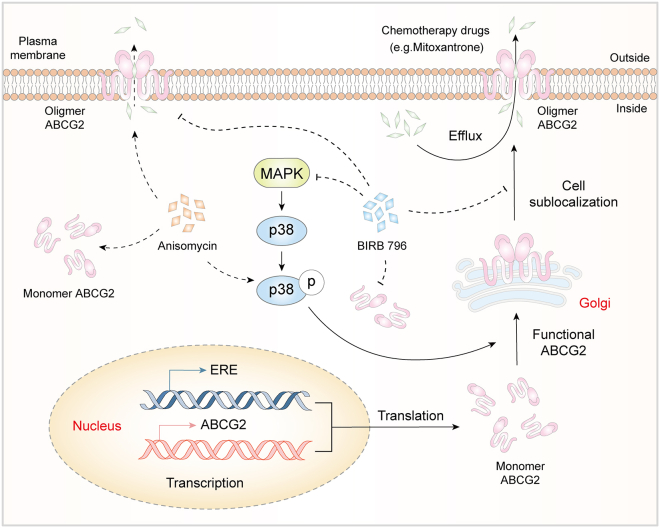
Schematic diagram of the mechanism of p38 regulating ABCG2-mediated decrease in chemotherapeutic sensitivity

To further elucidate the mechanisms regulating ABCG2’s efflux function, we conducted a comprehensive investigation. By using various databases and employing a p38 agonist and inhibitor, we confirmed that p38 modulates chemosensitivity in ABCG2-expressing cells. Inhibition of p38 increased the sensitivity of SiHa, MCF7, and MCF7/ADR cells to TP, MIT, and DOX, suggesting that p38 inhibitors might suppress ABCG2-mediated drug efflux independently of substrate binding sites. We also discovered that p38 modulated ABCG2 function by regulating its oligomer formation and membrane localization. Notably, the regulation of ABCG2 oligomerization by p38 exhibited a certain degree of cell type-specificity. In SiHa, MCF7/ADR, and MDA-MB-231 cells, p38 inhibition not only reduced the overall expression levels of both ABCG2 monomers and oligomers but also significantly decreased the oligomer-to-monomer ratio, suggesting that p38 influences both the abundance of ABCG2 and its oligomerization efficiency. However, a distinct pattern was observed in MCF7 cells. While p38 inhibition led to a coordinated reduction in the levels of both monomers and oligomers and caused a slight but statistically significant decrease in their ratio, p38 activation increased the expression of both forms without significantly altering the oligomer-to-monomer ratio (Figure 8). This asymmetric regulation implies that in MCF7 cells, the p38 pathway primarily modulates ABCG2 protein expression, whereas the oligomerization process itself may be constrained by other limiting factors or operate near its maximal capacity under basal conditions. This heterogeneity underscores the complexity of ABCG2 function and suggests that the combined therapeutic strategies involving p38 inhibitors may need to be tailored based on the specific regulatory network of different tumor types.

Moreover, our findings help clarify previously conflicting reports regarding the relationship between p38 and ABCG2.^19^^,^^30^^,^^31^ Instead of focusing solely on total ABCG2 expression, which may not consistently reflect functional output, we demonstrate that p38 critically influences the oligomeric state and membrane localization of ABCG2, providing a more direct link to its drug efflux capability. However, as chemotherapeutic agents such as DOX can be transported by multiple ABC transporters, we cannot fully exclude potential off-target effects of p38 on other efflux pumps like ABCB1. Our data, together with previous reports, suggest a broader regulatory network involving p38 and other pathways. For instance, we previously found that CPT inhibits ABCG2 oligomerization, yet it has also been shown to activate MAPK signaling in ERα-deficient breast cancer cells.^32^ We hypothesize that ERα status may modulate p38 activity and thereby influence ABCG2 function. Future studies should aim to dissect the interplay between p38 and other signaling pathways, particularly those involving ERα, and to evaluate the functional contributions of related ABC transporters. Such work will be essential to advance p38 inhibition as a tailored therapeutic approach for overcoming multidrug resistance.

In addition to drug efflux, many factors contribute to the decline in chemotherapeutic sensitivity, including apoptosis, autophagy, hypoxia, and DNA damage.^33^ Cell plasticity and cancer stem cells have recently emerged as new players in therapy evasion.^33^ Under chemotherapeutic stress, tumor cells may enter a reversible drug-tolerant state, or under persistent pressure, adopt long-term dormancy or irreversible resistance.^34^ The crosstalk between mechanical signaling and drug resistance allows tumor cells to survive chemotherapy, especially targeted therapies.^35^ Cells undergoing epithelial-mesenchymal transition often exhibit increased drug efflux pump gene expression, resistance to apoptosis, anoikis, and stem cell traits. Given this multifactorial nature of chemoresistance, simultaneously targeting several pathways may represent a more effective strategy than single-target approaches. Notably, beyond its role in modulating ABCG2 as shown in our study, p38 has been reported to participate in DNA damage repair, epithelial-to-mesenchymal transition, and ferroptosis resistance.^36^^,^^37^^,^^38^ These broad functional associations position p38 as a potential central node in the network of chemotherapy sensitivity regulation. However, just as the degree of chemoresistance reversal by the p38 inhibitor BIRB 796 varies among different cell lines and chemotherapeutic agents, the regulation of ABCG2 function by p38 may also be influenced by a variety of factors.

Our work establishes that p38 inhibition disrupts ABCG2 oligomerization, but the mechanistic details underlying p38-mediated regulation of ABCG2 oligomerization remain elusive. Key questions persist, such as whether p38 acts through ABCG2-interacting partners or via direct modification of functional residues. Previous studies have identified several structural determinants of ABCG2 assembly and localization. For instance, mutations in glycine residues at 406–410 and 553 impair ABCG2 function and trafficking,^39^^,^^40^ while the region spanning residues 528–655 is essential for homooligomerization.^41^ Although cysteine 603 and 608 are involved in the formation of ABCG2 dimers, cysteine-mediated covalent dimerization is not required for transport function.^42^ Phosphorylation represents another crucial regulatory layer. It is hypothesized to be a prerequisite for ABCG2 membrane expression.^43^^,^^44^ Tyrosine phosphorylation of ABCG2 facilitates its localization to the luminal membrane and inhibits drug absorption through its interaction with β-catenin.^43^ The 44 kDa serine/threonine kinase Pim-1 (Pim-1L) has been documented to protect prostate tumor cells from chemotherapy-induced apoptosis by phosphorylating ABCG2 at threonine 362.^44^ The integrity of this phosphorylation site is crucial for the subcellular distribution and multimerization of ABCG2. As a serine/threonine kinase, p38 may similarly regulate the phosphorylation, oligomerization and function of ABCG2. This hypothesis merits further investigation. Therefore, future studies should aim to identify whether p38 phosphorylates ABCG2 directly, which specific sites are involved, and whether other binding partners participate in this process. A deeper understanding of both the structural basis of ABCG2 oligomerization and its phosphorylation-mediated regulation will be essential to advance p38-targeted strategies for overcoming multidrug resistance.

### Limitations of the study

Although our study establishes the role of p38 in regulating ABCG2-mediated resistance, several limitations remain. The precise mechanism of how p38 disrupts ABCG2 oligomerization remains unclear, including whether it acts by interfering with specific phosphorylation sites or through intermediary proteins. Additionally, it is unknown whether p38 inhibition can sustain the reversal of ABCG2-mediated resistance long term without triggering compensatory upregulation of other efflux pumps.

## Resource availability

### Lead contact

Further information and requests for resources and reagents should be directed to and will be fulfilled by the lead contact, Wenxing Chen (chenwx@njucm.edu.cn).

### Materials availability

This study did not generate new unique reagents.

### Data and code availability

• All data in this paper will be shared by the lead contact upon request.
• This paper does not report original code.
• Any additional information required to reanalyze the data reported in this paper is available from the lead contact upon request.

## Acknowledgments

We acknowledge Jun Wang for the gift of the pCFP-ABCG2 plasmid and pYFP-ABCG2 plasmid. This project was supported in part by the 10.13039/501100001809National Natural Science Foundation of China (no. 82274150, no. 82374322, and no. 82304900).

## Author contributions

W.C. and Y.L. conceived and designed the experiments; Y.P., Z. Zhu, Y.Z., T.H., and H.F. performed the experiments; Z. Zhang and S.Y. analyzed the data; A.W. and Z.W. contributed to data curation, data analysis, and software; Y.P. wrote the paper. W.C. and Y.L. contributed to funding acquisition, supervision. W.C. reviewed and edited the manuscript. All co-authors have read and approved the final version of the manuscript.

## Declaration of interests

The authors declare no conflict of interest.

## STAR★Methods

### Key resources table

**Table:** 

REAGENT or RESOURCE	SOURCE	IDENTIFIER
**Antibodies**		

BCRP/ABCG2 antibody	Proteintech	Cat#10051-1-AP; RRID:AB_2220309
p38 MAPK Rabbit Monoclonal Antibody	Cell Signaling Technology	Cat#54470SF; RRID:AB_3719934
Phospho-p38 MAPK (Thr180/Tyr182) Rabbit Monoclonal Antibody	Cell Signaling Technology	Cat#4511T; RRID:AB_3719935
GAPDH polyclonal antibody	Bioworld Technology	Cat#AP0063; RRID:AB_2651132
Goat Anti-Rabbit IgG (H + L) HRP	Bioworld Technology	Cat#BS13278; RRID:AB_2773728
Goat anti-Rabbit IgG (H + L) Secondary Antibody, FITC	Thermo Fisher Scientific	Cat#65–6111; RRID:AB_2533966

**Chemicals, peptides, and recombinant proteins**		

Mitoxantrone	Meilunbio	MB1404
Topotecan	Sigma-Aldrich	T2705
Doxorubicin	Sigma-Aldrich	D1515
BIRB 796	MCE	HY-10320
Anisomycin	Cell Signaling Technology	#2222
BSA	Biosharp	BS114
3-(4,5-Dimethylthiazol-2-yl)-2,5-Diphenyltetrazolium Bromide(MTT)	BioFroxx	1334
RIPA lysis buffer	Dingguo Biotechnology	WB-0071
phenylmethylsulfonyl fluoride(PMSF)	Dingguo Biotechnology	WB-0181
phosphatase inhibitor cocktail	Beyotime Biotechnology	P1082
loading buffer	Beyotime Biotechnology	P0016
Dil	Beyotime Biotechnology	C1036

**Critical commercial assays**		

Membrane and Cytosol Protein Extraction Kit	Beyotime Biotechnology	P0033

**Experimental models: Cell lines**		

Human: MCF7	ATCC	HTB-22; RRID:CVCL_0031
Human: MDA-MB-231	Procell Life Science & Technology	CL-0150
Human: SiHa	Servicebio Technology	STCC10604P; RRID: CVCL_0032
Human: MCF-7/ADR	Shanghai Jinyuan Biotechnology	JY721

**Oligonucleotides**		

p38 shRNA	Corues Biotechnology	N/A

**Software and algorithms**		

ImageJ	NIH	https://imagej.nih.gov/ij/; RRID:SCR_003070

### Experimental model and study participant details

#### Cell culture

Human breast cancer cell lines MCF7, MDA-MB-231, and human cervical squamous cell line SiHa were obtained from the American Type Culture Collection (Manassas, VA, USA), the Procell Life Science & Technology Co., Ltd., and Servicebio Technology Co., Ltd (Wuhan, China), respectively. Doxorubicin multidrug-resistant cell line MCF-7/ADR was purchased from Shanghai Jinyuan Biotechnology Co., Ltd. RPMI-1640 Medium with 10% FBS was used to culture MCF7 cells while DMEM with 10% FBS was applied to incubate MDA-MB-231 cells. SiHa cells were cultured in MEM medium with 10% FBS and 1% NEAA, and MCF7/ADR cells were cultured in 1640 with 10% FBS and 500 ng/mL DOX. All of them were grown in 5% CO_2_ at 37°C. All cell lines used in this study were subjected to STR authentication and tested for the absence of mycoplasma infection.

### Method details

#### Non-reducing gradient gel electrophoresis

The non-reducing gradient gel electrophoresis was performed as described.^45^ The protein samples were denatured at 100°C for 3 min with a loading buffer (Beyotime Biotechnology, Shanghai, China) without reducing agents including DTT (Dithiothreitol) or 2-ME (2-Mercaptoethanol) and denaturing reagents such as SDS (Sodium dodecyl sulfate). The remaining steps were essentially similar to western blotting analysis. 6% SDS-PAGE gel and a multicolor broad-range protein ladder (10-260 kDa, Thermo Scientific, Waltham, MA, USA) were used when detecting the ABCG2 polymer.

#### Western blotting analysis

MCF7, MDA-MB-231, SiHa, and MCF7/ADR cells were collected and lysed in RIPA buffer with PMSF and phosphatase inhibitor cocktail. The protein samples were separated by 6%–10% SDS-PAGE gel and transferred to PVDF membranes. ABCG2 (Proteintech, Rosemont, USA), p38 MAPK (D13E1) XP, Phospho-p38 MAPK (Thr180/Tyr182) (D3F9) XP, GAPDH (Bioworld Technology, MN, USA) antibodies were used as primary antibodies. Goat Anti-Rabbit IgG(H + L) HRP (Bioworld Technology, MN, USA) was used as a second antibody. The signal was detected by the ChemiDoc XRS^+^ imaging system (Bio-Rad, Hercules, USA), and the results were quantified using gray value analysis by ImageJ software.

#### Cell viability assay

Cells were inoculated in a 96-well plate at a density of 1×10^4^/well. After treatment with different concentrations of drugs for 48h or 72 h, 0.5% MTT was added and incubated at 37°C for 4 h. The formazan was dissolved in DMSO and the optical density (OD) was measured at 490 nm using the BioTek Synergy2 microplate reader (BioTek Instruments, VT, USA).

#### Drug efflux experiment

The drug efflux experiment was performed as described.^14^ MCF7, MDA-MB-231, and MCF7/ADR cells were seeded in 6-well plates and treated with different concentrations of BIRB 796 or anisomycin. Then cells were collected by centrifugation and resuspended by serum-free medium. Cells except the blank group were treated with 10 μM MIT/DOX/TP and incubated at 37°C without light for 30 min 4°C, 1500 rpm/min, centrifuged for 5 min, discarded the supernatant, finally and resuspended cells in pre-cooled PBS. CytoFLEX (BECKMAN COULTER, USA) was used to determine the fluorescence accumulation of MIT/DOX/TP. The detection channel was FL-5/FL-2/FL-1. The Excitation/Emission wavelengths of MIT, DOX, and TP were 488/660 nm, 488/575 nm, and 488/525 nm, respectively.

#### Plasmids and transient transfection

The p38 shRNA (Forward: 5′-gatccgCTCGGCACACAGATGATGAAActcgagTTTCATCATCTGTGTGCCGAGtttttt-3′; Reverse: 5′-aattaaaaaaCTCGGCACACAGATGATGAAActcgagTTTCATCATCTGTGTGCCGAGcg-3′) was synthesized by Corues Biotechnology (Nanjing, China). SiHa cells (3×10^5^ cells/well) were planted in a 6-well plate. The p38 shRNA plasmid (400 ng/μL) was diluted in Opti-MEM and mixed with lipofectamine 2000 reagent (Life Technologies, NY, USA). After 6h transfection, the medium was changed to normal medium and sequentially incubated at 37°C for 16 h. The cells transfected with scramble control shRNA (shCON) were used as a negative control. The transfection was confirmed by q-PCR and western blot.

#### Fluorescence resonance energy transfer (FRET) microscopy imaging

The FRET was performed as previously described.^46^ The pCFP-ABCG2 plasmid and pYFP-ABCG2 plasmid were a gift from Jun Wang, Drug Discovery and Design Center, Shanghai Institute of Materia Medica, Chinese Academy of Sciences (Shanghai, China). The MCF7/ADR cells were seeded in a confocal culture dish (35 mm) and transfected when cell confluence reached 70%. 1 μg pCFP-ABCG2, 1 μg pYFP-ABCG2, and 3 μL lipofectamine 2000 reagent (Life Technologies, NY, USA) were mixed with 50 μL Opti-MEM medium, respectively. The plasmid and transfection reagent were incubated at room temperature for 5 min, and then lightly mixed and incubated for 10 min at room temperature. After adding 100 μL mixture dropwise to 1 mL serum-free medium and incubating for 6 h, another 16 h-incubation at 37°C was needed on the back of replenishing 100 μL FBS. The drug could be administrated followed by successful transfection. Leica inverted fluorescence microscope (Leica Microsystems, Solms, Germany) and ImageJ software were applied to collect and analyze the living cell FRET images, respectively. FRET efficiency is determined using the formula: FRET efficiency = (I_post_ - I_before_)/I_post_×100, where I_before_ and I_post_ represent the donor (pCFP) fluorescence intensity before and after photobleaching of the acceptor (pYFP), respectively.

#### Cell membrane protein and cytoplasmic protein extraction

Extraction of cell membrane protein and cytoplasmic protein according to the protocol of the Membrane and Cytosol Protein Extraction Kit (Beyotime Biotechnology, Shanghai, China). After 1h of administration of different concentrations of BIRB 796 or anisomycin, the cells were washed with ice PBS, scraped off with a scraper, and collected in a centrifuge tube at 4°C, 600g, 5 min. The PBS solution was discarded and the cell pellet was resuspended in Reagent A with PMSF (1:100 dilution), placed in an ice bath for about 10–15 min, freeze-thawed twice with liquid nitrogen to lyse cells, and then centrifuged at 4°C, 700g, for 10 min to discard the precipitate containing nuclei and unbroken cells. The supernatant was carefully collected, and centrifuged at 4°C, 14,000g, 30min. The supernatant was collected as cytoplasmic extracts. The pellet was resuspended in Reagent B, vortexed 5s, and kept in the ice bath for 5–10 min, and centrifuged at 14,000g, for 5min at 4°C. The final supernatant was collected cell membrane fraction. Both cytoplasmic and membrane extracts were stored at −80°C until use.

#### Immunofluorescent staining

Cells were seeded in advance into 6-well plates containing sterile coverslips and administered when there were sufficient cells on the coverslips. Staining was performed with 5 μmol/L DiI (Beyotime Biotechnology, Shanghai, China), a cell membrane dye, at 37°C for 15 min. Cells were then fixed with 4% paraformaldehyde for 20 min, blocked in 1% BSA for 1 h, and incubated with the corresponding primary antibody (1:100 dilution) overnight at 4°C. Goat anti-rabbit IgG H&L FITC (1:1000 dilution) and Hoechst nuclear dye were incubated for 2 h and 10 min against light, respectively. The images were taken by a laser scanning confocal microscope (Leica TCS SP5 X, Solms, Germany).

### Quantification and statistical analysis

Data are presented as mean ± SD. The sample size for each experiment is shown in the figure legends. The protein expression levels and fluorescence intensity were quantified by ImageJ software. Statistical significance was determined using Student’s *t* test for comparisons between two groups or one-way ANOVA for comparisons among more than two groups with GraphPad Prism 5.0. *p* < 0.05 was considered statistically significant.
