# Supplementary material for: p38 inhibition restores chemosensitivity of tumor cells by disrupting oligomerized breast cancer resistance protein membrane trafficking
Source: iScience. 2025 Dec 10;29(1):114359. doi: 10.1016/j.isci.2025.114359 (PMC12800634; doi:10.1016/j.isci.2025.114359)
Supplement: Document S1. Figures S1–S4, Table S1 and S2 [file mmc1.pdf]

iScience, Volume *29*

Supplemental information

p38 inhibition restores chemosensitivity of tumor cells by disrupting oligomerized breast cancer resistance protein membrane trafficking

Yanhong Pan, Ziyan Zhu, Yunxuan Zhu, Tongyao Hu, Zhengyu Zhang, Hui Fan, Suyun Yu, Zhonghong Wei, Aiyun Wang, Yin Lu, and Wenxing Chen

Supplementary information

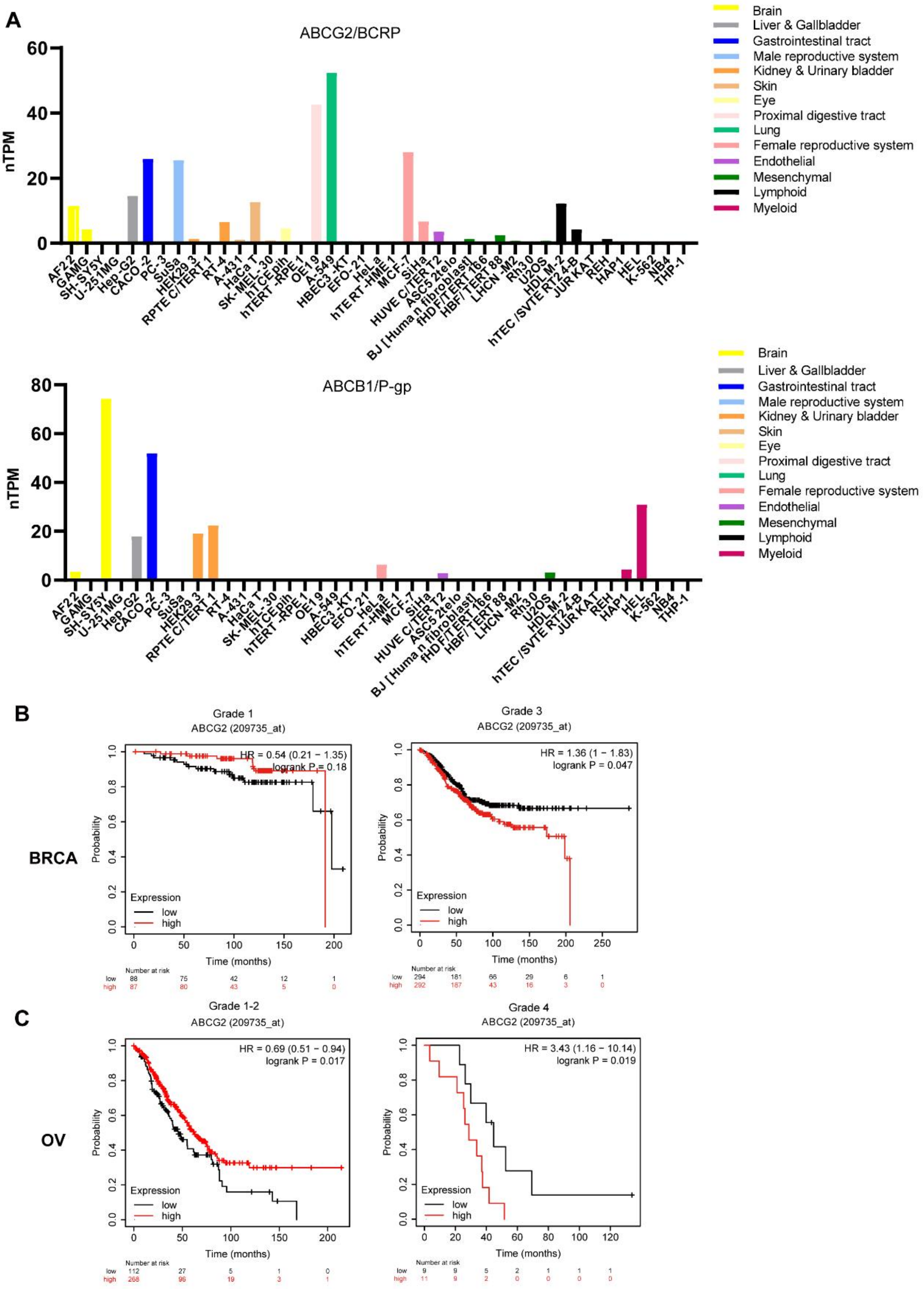

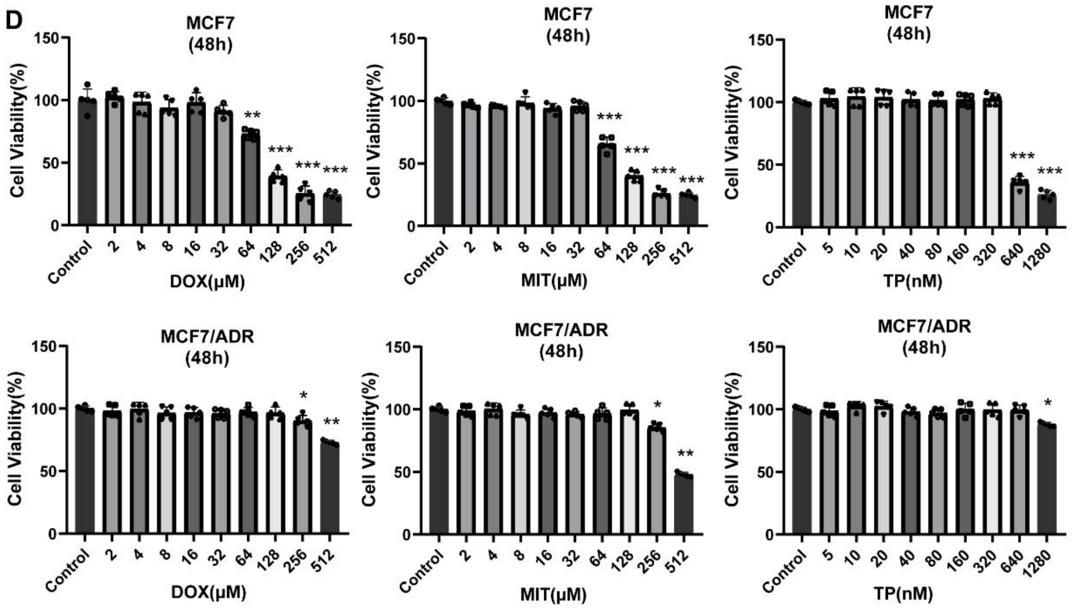

Figure S1 ABCG2 is involved in decreased sensitivity of tumor cells to chemotherapy

(A) The mRNA expression of ABCG2 and ABCB1 in different cells. The resources were acquired from the HPA database. (B-C) The ABCG2-related overall survival probability of patients with BRCA or OV of different stages. The clinical data was acquired from the Kaplan-Meier Plotter database. (D) MCF7/ADR cells and MCF7 cells treated with DOX, MIT, and TP at various concentrations for 48h. The Cell viability was measured using MTT assay. n =5 for each group. Data are mean ± SD, *P<0.05, **P<0.01, ***P<0.005.

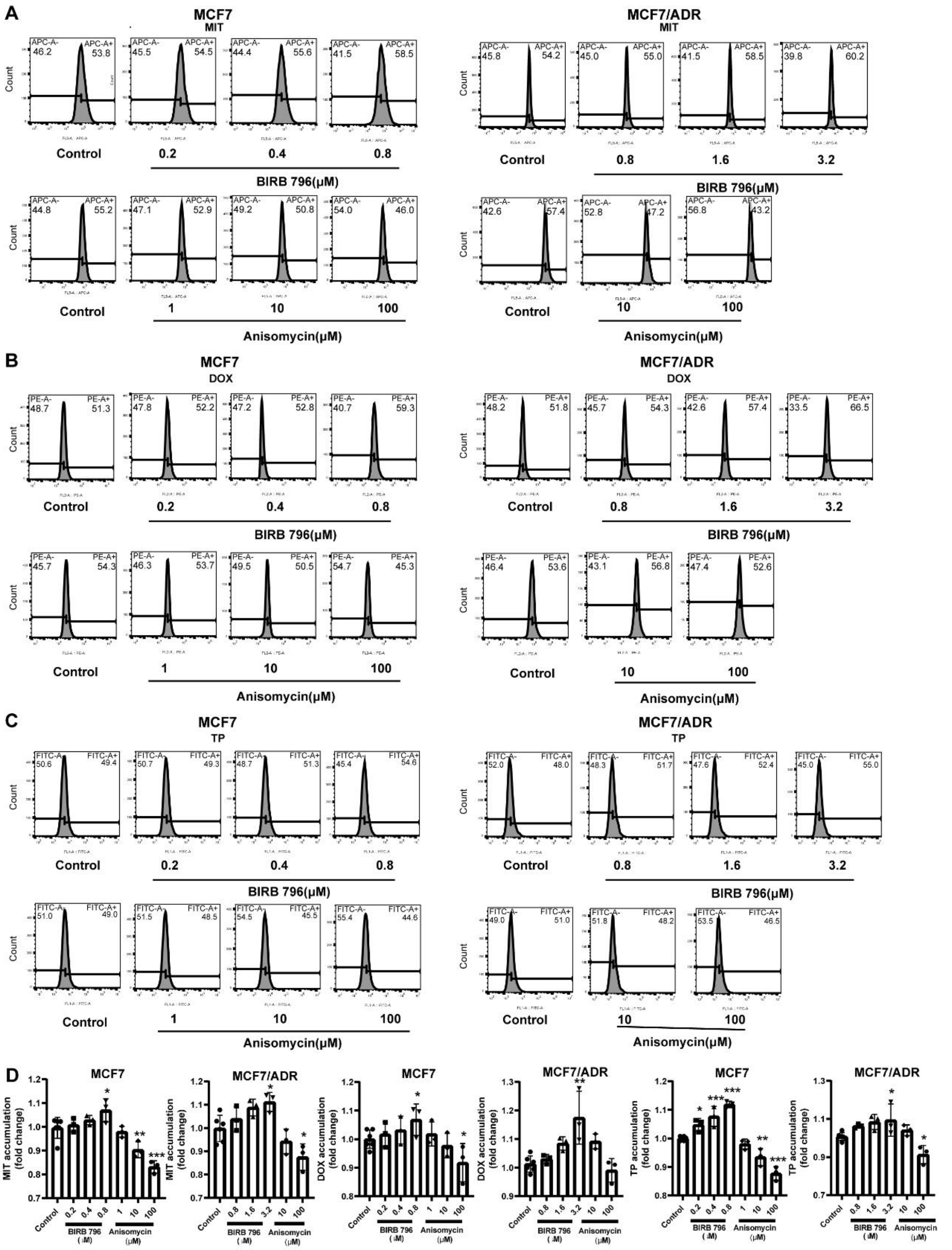

Figure S2 The efflux of tumor cells to chemotherapeutic drugs is regulated by p38

MCF7 cells and MCF7/ADR cells were treated with MIT(A), DOX (B), or TP (C) after 1h of use of anisomycin or BIRB 796. The drug fluorescence accumulation was

detected by flow cytometry. These quantitated results are shown in (D). n=6 for control group. n=3 for other groups. Data are mean ± SD, *P<0.05, **P<0.01, ***P<0.005.

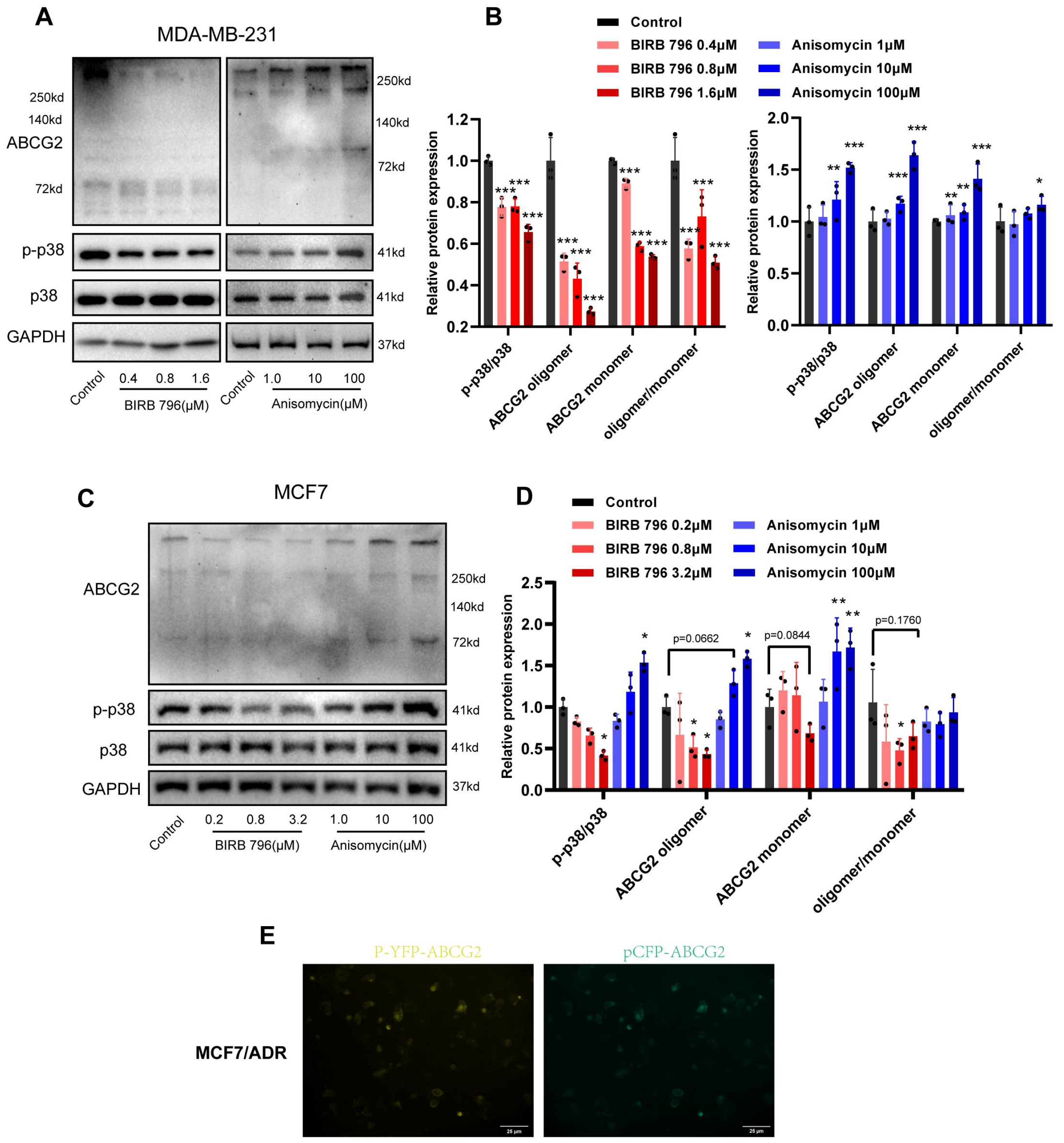

Figure S3 The formation of ABCG2 oligomer was suppressed by the p38 inhibitor

(A-D) The protein levels of p-p38, p-38, ABCG2 monomer, and ABCG2 oligomer in MDA-MB-231 and MCF7 cells treated with BIRB 796 or anisomycin. The quantitative analysis of protein expression is shown in (B, D). n=3 for each group. (E) MCF7/ADR cells were transfected with pCFP-ABCG2 and pYFP-ABCG2 plasmids, respectively. The cells were observed microscopically for ABCG2 expression. Scale bar: 25 μm. n=3 for each group. Data are mean ± SD, *P<0.05, **P<0.01, ***P<0.005.

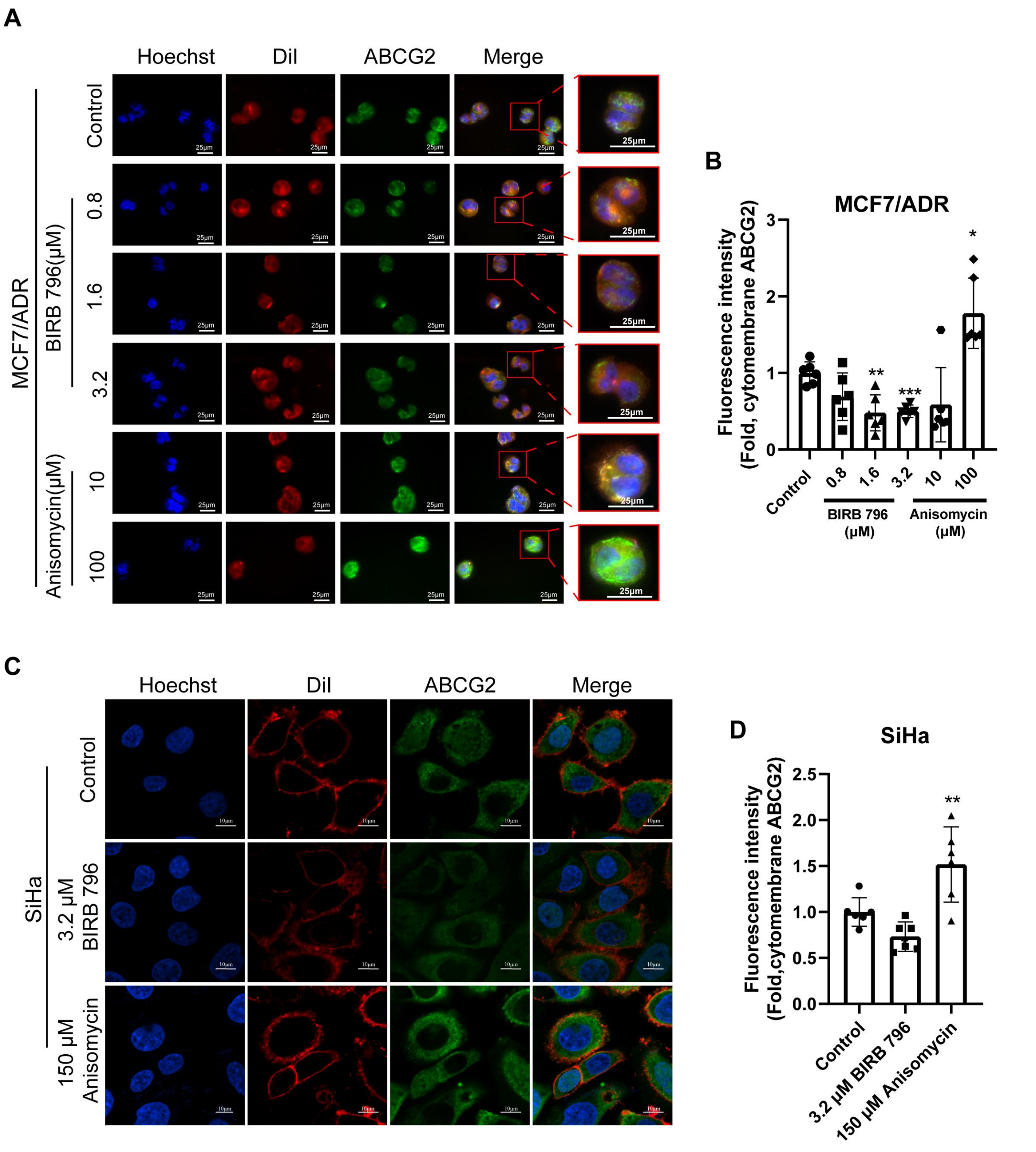

Figure S4 Adjusting the activation of p38 regulates the membrane localization of ABCG2

Immunofluorescence staining and quantitative analysis of fluorescence intensity for ABCG2 in MCF-7/ADR (A, B) and SiHa (C, D) cells treated with BIRB 796 or anisomycin for 1h. Dil was used to indicate cell membrane. (A) The representative images of ABCG2 in MCF-7/ADR cells. Scale bar: 25 μm. (C) The representative

images of ABCG2 in SiHa cells. Scale bar: 10 μm. n = 6 for each group. Data are mean ± SD, *P<0.05, **P<0.01, ***P<0.005.

Table S1 The relevance scores of the genes shared among multidrug resistance, MIT sensitivity, DOX sensitivity, and TP sensitivity

	Multidrug resistance	DOX sensitivity	MIX sensitivity	TP sensitivity
ABCB1	94.64163208	41.2130394	28.85347366	25.86048126
ABCC1	73.85743713	24.58073425	19.70965195	16.32028961
ABCG2	48.79921722	18.7684288	32.63182831	19.82862091
TP53	37.31440353	39.48664093	25.29332733	30.6981926
EGFR	30.74138069	24.666996	16.56806946	24.74414635
IL6	30.91107559	21.90496254	16.53548622	16.05518532

The data were required from the Genecards database.

Table S2 The correlation analysis of ABCG2 and its interacting MAPK pathway-related genes

	BRCA		CESE		OV		UCEC		UCS	
	P value	R	P value	R	P value	R	P value	R	P value	R
DUSP10	0.77	0.0088	0.98	-0.0012	0.84	-0.0099	0.67	-0.033	0.68	-0.056
FGR	0.092	0.051	6.3e−05	0.23	0.65	0.022	3e−05	0.31	0.28	0.15
MAP2K1	0.15	0.044	0.38	-0.05	0.026	0.11	0.8	-0.019	0.34	0.13
MAP2K3	0.12	-0.047	0.62	-0.028	0.00028	0.18	0.41	-0.063	0.82	0.031
MAP2K6	0.19	0.04	0.44	0.044	0.035	0.1	0.97	-0.0031	0.61	0.07
MAPK14	2.6e−05	0.13	0.00034	0.2	2.8e−12	0.33	0.014	0.19	0.022	0.3
MAPKAPK2	0.4	-0.026	0.38	0.051	9.1e−08	0.26	0.97	0.0033	0.39	0.12
MAPKAPK3	0.31	0.0031	0.77	0.017	2.6e−06	0.23	0.45	-0.057	0.21	0.17
MKNK1	0.06	0.057	0.031	0.12	1.8e−05	0.21	0.44	0.059	0.63	0.065
MKNK2	0.54	0.019	0.034	-0.12	0.0083	0.13	0.16	-0.11	0.15	-0.2
P2RY1	3.7e−07	0.15	0.13	0.086	5.8e−07	0.24	1.9e−12	0.5	0.032	0.28
PPP1R15A	0.03	0.066	0.11	-0.091	0.25	0.055	0.58	0.043	0.24	-0.16
PTPN7	0.73	-0.011	0.0047	0.16	0.14	0.072	0.75	-0.024	0.31	0.14
PTPRR	0.52	0.02	0.31	-0.058	0.49	0.033	0.068	-0.14	0.86	-0.023

RPS6KA4	0.13	-0.046	0.28	-0.062	0.021	0.11	0.75	-0.024	0.38	0.12
RPS6KA5	0	0.26	0.0032	0.17	9.9e−09	0.27	0.11	0.12	0.0068	0.35
SLC22A2	0.47	0.022	0.22	0.07	0.32	0.049	0.1	0.12	0.9	-0.017
SLC22A3	0.052	0.059	0.96	0.0031	0.0028	0.14	0.0096	0.2	0.57	0.077
TAB1	0.54	0.019	1e−05	0.25	5.3e−05	0.19	0.0099	0.2	0.018	0.31

Data are obtained from GEPIA Database.
